# Evaluation of potential underuse of cardiac resynchronization therapy for heart failure with reduced ejection fraction

**DOI:** 10.1002/joa3.12647

**Published:** 2021-10-13

**Authors:** Makoto Takano, Yui Nakayama, Hisao Matsuda, Tomoo Harada, Yoshihiro J. Akashi

**Affiliations:** ^1^ Division of Cardiology Department of Internal Medicine Yokohama City Seibu Hospital St. Marianna University School of Medicine Yokohama Yokohama City Japan; ^2^ Division of Cardiology Department of Internal Medicine St. Marianna University School of Medicine Kawasaki Japan

**Keywords:** cardiac resynchronization therapy, guideline, heart failure, implantable cardioverter defibrillator, left bundle branch block

## Abstract

**Background:**

The number of patients with chronic heart failure is increasing in Japan. However, the annual number of patients with heart failure who receive cardiac resynchronization therapy (CRT) has been constant in the last few years. In this study, we evaluated patients who did not receive CRT despite being eligible for this treatment to elucidate the clinical impact of CRT administration.

**Methods:**

We assessed 214 patients with a left ventricular ejection fraction (LVEF) ≤ 50% (excluding patients treated with CRT) who underwent transthoracic echocardiography between January and May 2020 at our institution. The patients were stratified into two groups: Group A (n = 26; patients eligible for CRT) and Group B (n = 188; patients ineligible for CRT); however, all patients only received pharmacological therapy. We retrospectively analyzed the prognosis of these patients with respect to the cumulative number of hospitalizations for heart failure and cardiogenic deaths.

**Results:**

We observed no significant between‐group differences in age, sex, and severity/diagnosis of organic heart disease. Group A had a significantly higher number of hospitalizations for heart failure and cardiogenic deaths than Group B (log‐rank test, *P* < .01; hazard ratio, 3.05; 95% confidence interval, 1.31‐7.09; average follow‐up period, 675 days).

**Conclusions:**

This study shows that 12% of patients were eligible for CRT. However, the implantation rate was low and no one was implanted. CRT is underutilized in patients who have heart failure with reduced LVEF. Therefore, we strongly recommend CRT for patients with indications for CRT.

## Graphical abstract

This study evaluates the underutilization of cardiac resynchronization therapy in eligible patients with heart failure with a left ventricular ejection fraction less than 50%.

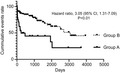

## 1 INTRODUCTION

There are over one million patients with heart failure in Japan, which is indicative of a “heart failure pandemic” in the country.^1^ About 250 000 patients (25% of patients) with heart failure have been hospitalized and have received medical treatment at least once, and after discharge, they have been treated as outpatients.^2^ However, the chronic nature of the disease results in a gradual deterioration in the condition and functioning of the heart, which necessitates repeated hospitalizations and eventually leads to death.

The Japanese Circulation Society (JCS)/The Japanese Heart Rhythm Society (JHRS) 2018 practical guidelines classify treatment regimens for progressive heart failure according to the patient's left ventricular ejection fraction (LVEF).^3^ The categories are as follows: heart failure with preserved ejection fraction (LVEF ≥ 50%), heart failure with midrange reduced ejection fraction (LVEF ≥ 40% and <50%), and heart failure with reduced ejection fraction (HFrEF) (LVEF < 40%). Treatment may be in the form of either pharmacological or non‐pharmacological management. According to the JCS/JHFS 2018 guidelines, pharmacological management for patients with HFrEF comprises standard drug therapies, such as β‐blockers. Non‐pharmacological management, primarily administered to patients with HFrEF, includes cardiac resynchronization therapy (CRT) and the implantation of devices, such as an implantable cardioverter defibrillator (ICD) to improve cardiac function and terminate ventricular arrhythmias, if it occurs, in order to prevent sudden cardiac death. However, in Japan, non‐pharmacological management is performed as per the JCS/JHS 2018 guidelines but the guidelines are underutilized in patients with heart failure.^4^

In this study, we attempted to investigate whether non‐pharmacological management, specifically CRT, is actually underutilized in patients with HFrEF in order to encourage the appropriate use of CRT according to the guidelines. Furthermore, we evaluated the prognosis of patients who were eligible for CRT but did not receive CRT at our institution.

## 2 METHODS

### 2.1 Patient selection and study design

**FIGURE 1:**
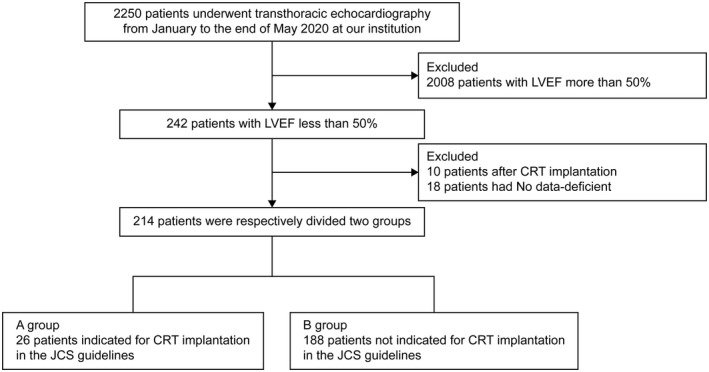
Description of the study protocol. Among patients who underwent transthoracic echocardiography between January and the end of May 2020 at our institution, this retrospective study assessed only patients with LVEF < 50% (242 patients) and excluded patients with LVEF > 50% (2008 patients). The final 214 patients were divided into two groups

Among patients who underwent transthoracic echocardiography between January and the end of May 2020 at our institution, this retrospective study only assessed patients with an LVEF of <50% (242 patients) and excluded patients with an LVEF of ≥50% (2008 patients). Among the 242 patients whose attending physician was a cardiologist, we excluded those who underwent CRT implantation (10 patients) or those for whom lack relevant data on echocardiography were unavailable (18 patients). The final 214 patients were divided into two groups. Group A consisted of 26 patients for whom CRT implantation was recommended as per the JCS/JHRS guidelines (2018 revised edition).^3^ Group B consisted of 188 patients for whom CRT implantation was not recommended as per the JCS/JHRS guidelines. The study design is summarized in Figure 1. From the time of the first recording of LVEF < 50% in each patient, hospitalization for heart failure and all‐cause mortality were retrospectively observed and compared.

The study protocol was approved by the Institutional Committee on Human Research at St. Marianna University School of Medicine (no. 4911, UMIN000041212), and consent was obtained from all patients (via the opt‐out method) at our institution.

### 2.2 Statistical analyses

Statistical analyses were performed using SPSS version 21.0 (IBM). Categorical comparisons between the groups were performed using a chi‐square test and a Fisher's exact test for independence. Continuous variables were compared using a Wilcoxon rank–sum test and a Mann–Whitney rank–sum test. All continuous parameters are presented as mean ± SD values. Differences were considered statistically significant if the *P*‐value was less than .05. Logit transformations of sensitivity and specificity were assumed by the bivariate approach used in this study.

## 3 RESULTS

Table 1 shows the baseline characteristics of the 214 patients. All of the patients were treated with optimal standard drug therapy according to the JCS/JHRS guidelines. However, although there were 26 (12.1%) patients who were indicated for CRT among all the patients who were treated with optimal standard drug therapy, CRT was not implanted.

**TABLE 1:** Baseline characteristics

	Patients with reduced EF (<50%) (n = 214)	Patients indicated and under‐treated for CRT (n = 26)	Patients not indicated for CRT (n = 188)	*P* value
Demographics				
Males, n (%)	159 (74.3%)	18 (69.2%)	141 (75.0%)	.53
Age (y)	70	76	71	.07
Height (cm)	163	165	165	.43
Body weight (kg)	64	61	63	.25
BMI (kg/m^2^)	24	23	24	.49
Cause of HF				
Ischemia, n (%)	64 (29.9%)	9 (34.6%)	55 (29.3%)	.65
Non‐ischemia, n (%)	115 (53.7%)	11 (42.3%)	104 (55.4%)	.29
Valvular, n (%)	18 (8.4%)	3 (11.5%)	15 (8.0%)	.47
Other, n (%)	17 (7.9%)	3 (11.5%)	14 (7.4%)	.45
Medical history				
Atrial fibrillation, n (%)	58 (27.1%)	6 (23.1%)	52 (27.7%)	.81
Hypertension, n (%)	124 (57.9%)	18 (69.2%)	124 (66.0%)	.83
Diabetes, n (%)	43 (20.1%)	4 (15.4%)	39 (20.7%)	.61
Dialysis, n (%)	5 (2.3%)	1 (3.8%)	4 (2.2%)	.48
Clinical				
NYHA class				
Ⅱ, n (%)	140 (65.4%)	18 (69.2%)	122 (64.9%)	.83
Ⅲ, n (%)	62 (29.0%)	6 (23.0%)	56 (29.8%)	.64
Ⅳ, n (%)	12 (5.6%)	2 (7.7%)	10 (5.3%)	.65
Heart rate (beats/min)	76 ± 16	77 ± 20	70 ± 21	.05
QRS width (ms)	107 ± 25	155 ± 23	101 ± 17	<.001
QRS ≥ 150 ms, n (%)	16 (7.5%)	11 (42.3%)	5 (2.7%)	<.001
150 ms > QRS ≥ 120 ms, n (%)	29 (13.6%)	15 (57.7%)	14 (7.4%)	<.001
120 ms > QRS, n (%)	169 (79.0%)	0 (0.0%)	169 (89.9%)	.14
LBBB, n (%)	13 (6.1%)	13 (50.0%)	0	
LVEF (%)	38 ± 8.5	35 ± 9.5	39 ± 8.2	.03
50% ≥ LVEF > 40%, n (%)	107 (50.0%)	9 (34.6%)	98 (52.2%)	.20
40% ≥ LVEF > 35%, n (%)	38 (17.8%)	4 (15.4%)	34 (18.1%)	>.99
35% ≥ LVEF, n (%)	69 (32.2%)	13 (50.0%)	56 (29.8%)	.05
Medications				
ACEI/ARB	204 (95.3%)	25 (96.2%)	179 (95.2%)	>.99
Beta‐blockers	193 (90.2%)	24 (92.3%)	169 (89.9%)	>.99
MRA	125 (58.4%)	16 (61.5%)	109 (58.0%)	.83
Diuretics	163 (76.2%)	20 (76.9%)	143 (76.1%)	>.99
Amiodarone	15 (7.0%)	2 (7.7%)	13 (6.9%)	>.99

Abbreviations: ACEI, angiotensin‐converting enzyme inhibitor; ARB, angiotensin‐receptor blocker; BMI, body mass index; CRT, cardiac resynchronization therapy; EF, ejection fraction; HF, heart failure; LBBB, left bundle branch block; LVEF, left ventricular ejection fraction; MRA, mineralocorticoid‐receptor antagonist; NYHA, New York Heart Association; QRS, xxx.

We found no significant between‐group differences in terms of age, sex, and severity/diagnosis of organic heart disease. Compared to Group B, Group A had a wider QRS complex (Group A vs Group B: QRS width, 155 ± 23 ms vs 101 ± 11 ms; *P* < .001), and a lower LVEF (Group A vs Group B: LVEF, 35 ± 9.5% vs 39 ± 8.2%; *P* = .03). This QRS is the QRS width of the electrocardiogram.

In Group A, 13 patients had typical LBBB and were hospitalized due to heart failure; among these, 2 patients died due to sudden cardiac death as a complication of ventricular fibrillation. The clinical courses of these 13 patients are presented in Table 2.

**TABLE 2:** Clinical course of patients with typical LBBB

Patients no	Age (y)	Sex	QRS width (ms)	LVEF (%)	Organic heart disease	HF hospitalization/cardiac death
1	48	Female	132	45	NICM	HF hospitalization
2	90	Female	132	42	NICM	HF hospitalization
3	84	Female	138	35	NICM	HF hospitalization
4	69	Male	138	38	DCM	HF hospitalization
5	88	Female	140	35	ICM	HF hospitalization
6	80	Female	142	46	NICM	HF hospitalization
7	76	Male	144	46	NICM	Death (VF)
8	88	Female	146	34	ICM	HF hospitalization
9	88	Female	146	34	ICM	HF hospitalization
10	86	Female	148	32	VHD	HF hospitalization
11	76	Female	148	32	NICM	HF hospitalization
12	78	Male	156	40	ICM	HF hospitalization
13	68	Male	196	35	DCM	Death (VF)

Abbreviations: DCM, dilated cardiomyopathy; HF, heart failure; ICM, ischemic cardiomyopathy; LBBB, left bundle branch block; LVEF, left ventricular ejection fraction; NICM, non‐ischemic cardiomyopathy; VF, ventricular fibrillation; VHD, valvular heart disease.

**FIGURE 2:**
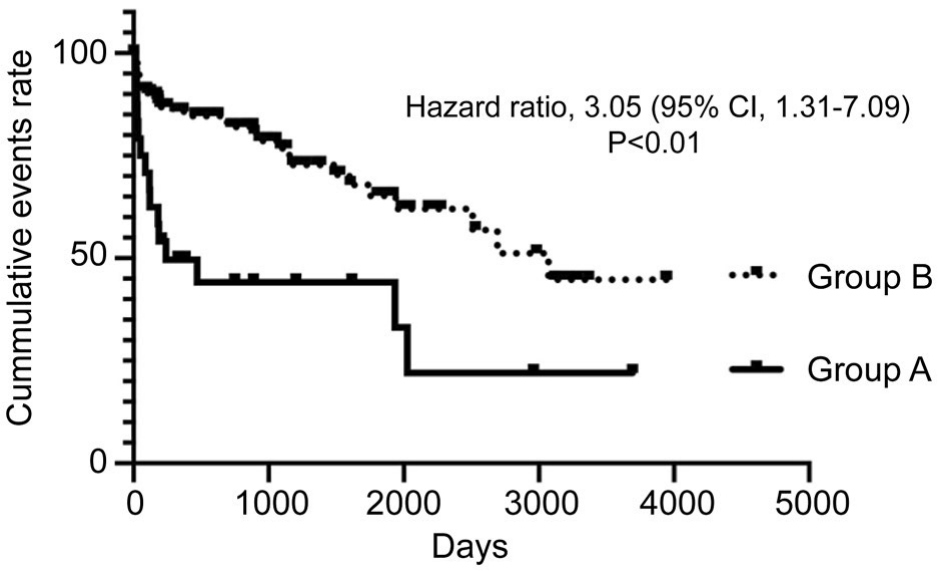
Time‐to‐event curve depicting hospitalizations for heart failure and cardiac deaths. The numbers of hospitalizations and cardiac deaths were greater in Group A (14 hospitalizations; 3 cardiac deaths) than in Group B (36 hospitalizations; 2 cardiac deaths) (log‐rank test, *P* < .01; hazard ratio, 3.05; 95% confidence interval, 1.31‐7.09; average follow‐up period, 675 d)

We retrospectively analyzed the two groups with respect to the number of hospitalizations for heart failure and cardiac deaths. The number of hospitalizations and cardiac deaths were greater in Group A than in Group B (log‐rank test, *P* < .01) (Figure 2).

## 4 DISCUSSION

This study assessed the prevalence and consequences of underutilization of CRT in patients with an LVEF of <50%. We observed that 12% of the patients (26 patients) with an LVEF of <50% had an interventricular conduction disorder. According to the JCS/JHRS guidelines, these patients should have received treatment along with CRT; however, they received only pharmacological management and showed poor clinical prognosis.

With reference to the IMPROVE‐HF (Registry to Improve the Use of Evidence‐Based Heart Failure Therapies in Outpatient Setting), the number of devices implanted in patients was lower than the number of eligible patients (ICD/CRT 51% and CRT: 39%).^4^

In Japan, only 10% of patients with HFrEF are treated with CRT, as reported by the Chronic Heart Failure Analysis and Registry in Tohoku District‐2 (CHART‐2) study.^5^ The CHART‐2 study also reported a significantly higher prevalence of fatal arrhythmic events in patients with an LVEF of ≤35% than in patients with an LVEF of >35%. Only 2.6% of patients underwent ICD implantation or CRT in the CHART‐2 study.^5^ The findings of our study, with reference to the underutilization of CRT, were consistent with those of CHART‐2. The progression of heart failure (stage) was greater among under‐treated patients with HFrEF who were eligible for CRT than among those not eligible for CRT. Furthermore, our study revealed that CRT‐eligible patients had poorer prognosis compared to those patients who were not eligible for CRT.

Cardiac resynchronization therapy administration and ICD implantation are reportedly low in Western countries.^6^, ^7^ Recently, underutilization of CRT in Europe has also been reported.^8^ Some studies have assessed the factors influencing the low rate of CRT administration and ICD implantation in Western countries; however, no such study has been conducted in Japan. This study presents the underutilization of non‐drug treatment in Japan. Hence, the following treatment plans may be beneficial. A specific treatment plan was mentioned in the study by Gravelin et al and their report can be used to standardize the indication of non‐drug treatment using a screening tool that evaluates LVEF, and such a screening tool can be used to treat heart failure.^9^ According to a report by Sadarmin et al, in order to avoid underutilization of non‐drug treatment, it is necessary for a cardiologist to consult with an electrophysiologist regarding non‐pharmacological treatment.^10^ In the present study, 12% of patients with an LVEF of <50% were eligible for CRT according to the JCS/JHRS guidelines; however, they were not offered this treatment. One reason cited for not implementing CRT treatment was the difficulties caused by coronary venous anatomy. In our institution, the success rate of LV lead implantation is 98%. Conversely, 2% of patients have unsuccessful LV lead implantation. Some physicians did not opt for CRT due to inadequate experience or training regarding left ventricular lead implantation. The lack of proper patient screening and the lack of patient referral may also account for the underutilization of CRT in Japan. Another reason why CRT may not have been applied to patients was that the physicians may not have been aware of the indications of CRT.

This study had some limitations. First, it retrospectively analyzed a small number of patients from a single center. Therefore, further prospective studies with larger cohorts are warranted. Second, both groups do not include patients with a history of ventricular arrhythmia who had received secondary preventive measures. Furthermore, CRT‐eligible patients included those with atrioventricular conduction disorders who may require right ventricular pacing; therefore, future studies should evaluate the prevalence and risk stratification of heart failure in patients with various structural heart diseases.

Heart failure is a progressive disease, and there may be some patients whose eligibility for CRT may change during the clinical course. Additionally, patients in Group A were older than those in Group B, which may be why CRT was not administered to patients in Group A.

## 5 CONCLUSIONS

According to the JCS guidelines, CRT is recommended for patients with HFrEF with an intraventricular conduction disorder. However, only 12% of patients were eligible for CRT, and the implantation rate is low compared to the reality that no one was implanted. CRT is underutilized in patients who have heart failure with reduced LVEF. CRT is recommended for eligible patients with heart failure to improve their prognosis.

## CONFLICT OF INTEREST

Authors declare no Conflict of Interests for this article.

## Data Availability

Not applicable.
